# Resistance of bone marrow stroma to genotoxic preconditioning is determined by p53

**DOI:** 10.1038/s41419-021-03824-3

**Published:** 2021-05-26

**Authors:** Natalia Fedtsova, Elena A. Komarova, Kellee F. Greene, Liliya R. Novototskaya, Ivan Molodtsov, Craig M. Brackett, Evguenia Strom, Anatoli S. Gleiberman, Alexander N. Shakhov, Andrei V. Gudkov

**Affiliations:** 1 Department of Cell Stress Biology, Roswell Park Comprehensive Cancer Center, Buffalo, NY 14263 USA; 2 Gamaleya National Research Center of Epidemiology and Microbiology, Moscow, Russia; 3 Everon Biosciences, LLC., Buffalo, NY 14203 USA; 4 Genome Protection Inc, Buffalo, NY 14203 USA; 5 Buffalo Biolabs., LLC, Buffalo, NY 14203 USA

**Keywords:** Haematopoietic stem cells, Stem-cell research

## Abstract

Transplantation of bone marrow (BM) is made possible by the differential sensitivity of its stromal and hematopoietic components to preconditioning by radiation and/or chemotherapeutic drugs. These genotoxic treatments eliminate host hematopoietic precursors by inducing p53-mediated apoptosis but keep the stromal niche sufficiently intact for the engraftment of donor hematopoietic cells. We found that p53-null mice cannot be rescued by BM transplantation (BMT) from even the lowest lethal dose of total body irradiation (TBI). We compared structural changes in BM stroma of mice differing in their p53 status to understand why donor BM failed to engraft in the irradiated p53-null mice. Irradiation did not affect the general structural integrity of BM stroma and induced massive expression of alpha-smooth muscle actin in mesenchymal cells followed by increased adiposity in p53 wild-type mice. In contrast, none of these events were found in p53-null mice, whose BM stroma underwent global structural damage following TBI. Similar differences in response to radiation were observed in in vitro-grown bone-adherent mesenchymal cells (BAMC): p53-null cells underwent mitotic catastrophe while p53 wild-type cells stayed arrested but viable. Supplementation with intact BAMC of either genotype enabled donor BM engraftment and significantly extended longevity of irradiated p53-null mice. Thus, successful preconditioning depends on the p53-mediated protection of cells critical for the functionality of BM stroma. Overall, this study reveals a dual positive role of p53 in BMT: it drives apoptotic death of hematopoietic cells and protects BM stromal cells essential for its functionality.

## Introduction

Bone marrow transplantation (BMT) is a curative therapy for a variety of diseases including hematological disorders, immune deficiencies, and solid tumors^1–3^. BMT includes the elimination of the diseased hematopoietic system through a course of chemotherapy and/or irradiation, termed preconditioning, followed by transplantation of healthy donor bone marrow (BM) containing self-renewing multipotent hematopoietic stem and progenitor cells (HSC/HPC)^4,5^.

BMT relies on differential sensitivity of BM stromal and hematopoietic cellular components to radiation or chemotherapy that are used for preconditioning. Hematopoietic cells are highly radiosensitive via the mechanism that involves p53-mediated apoptosis^6,7^. On the contrary, stromal cells survive doses of irradiation that kill the hematopoietic system^8–13^, enable engraftment of donor hematopoietic precursors, and re-constitution of the hematopoiesis.

A regulatory role in creating a niche for HSC/HPC and their maintenance initially was attributed to bone-forming osteoblasts^14,15^. However, recent studies revealed that HSCs reside in perisinusoidal rather than in endosteal (osteoblastic) niches^16–20^. Several candidate niche cell types, including both non-hematopoietic (e.g., perivascular mesenchymal stem and endothelial cells) and HSC-derived (e.g., megakaryocytes, macrophages) were identified^21^. Mesenchymal stromal cell (MSC) subtypes were identified by expression of Leptin receptor (Lepr)^17,18^, Nestin (Nes)^22^, or NG2 (Cspg4)^23^. Most of them produce key HSC niche factors^19^ and are capable of differentiation to cells of the osteogenic lineage, adipocytes, and chondrocytes^7,16,17,19,22,24–26^. Recent studies revealed high cell heterogeneity within BM stromal niche cells. Baryawno et al.^27^ using single-cell RNA sequence analysis defined 17 distinct cell subsets with new mesenchymal, pericyte, fibroblast, and endothelial subpopulations among Lepr, Nestin, and NG2-expressing cells. Tikhonova et al.^28^ determined different cell clusters within the LepR+ compartment (adipogenesis-associated and osteogenesis-associated) and the formation of a novel adipo-primed cell cluster after stress (5-FU) that correlated with the expansion of adipocytes following BM insult shown before^29,30^.

This study was triggered by the observation that p53-null mice exposed to lethal doses of total body irradiation (TBI) could not be rescued by BMT, which was highly efficient in p53 wild-type mice subjected to the same TBI doses. Trying to understand this phenomenon, we found cardinal differences in the degrees of damage caused by radiation preconditioning in the stromal components of wild-type and p53-null mice. We demonstrated that functional p53 protects stromal cells in irradiated BM from IR-induced mitotic catastrophe, which is the cause of death of p53-deficient cells. We provided functional proof for our model by demonstrating partial restoration of donor BM engraftment and extending the life of irradiated p53-null mice by supplementing donor BM with bone-derived adhesive mesenchymal cells (BAMC).

## Results

### BMT fails to rescue lethally irradiated p53-null mice

**Fig. 1:**
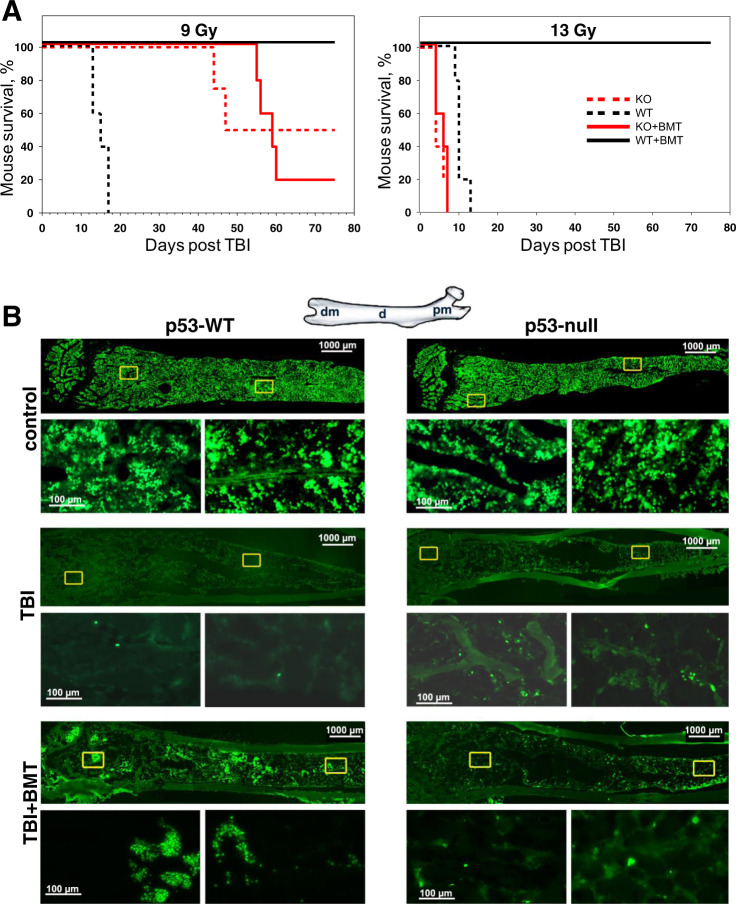
Successful BMT after IR preconditioning in mice requires p53 expression by the host. **A** BMT fails to rescue lethally irradiated p53-null mice. Groups of p53-knockout (p53-KO) and p53-WT male C57BL/6 mice were irradiated with lethal doses of TBI (9 or 13 Gy; *n* = 7–10 mice/group at each TBI dose) without or with transfusion of p53-WT BM cells (5 × 10^6^ cells/mouse; BMT) 24 h later. The survival of mice was monitored daily for 60–75 days. Representative results from one of three independent experiments are shown. The difference in survival between p53-WT and p53-KO mice transplanted with p53-WT BM was statistically significant (*P* < 0.01 by two-tailed Fisher’s exact test) beginning 7 and 60 days after 13 and 9 Gy TBI, respectively. **B** After IR and BMT, clusters of EdU+ proliferating cells are present in the BM of p53-WT mice but not in the BM of p53-null mice. Longitudinal sections of femoral BM from p53-WT and p53-null mice were stained to detect EdU incorporation (green fluorescence). p53-WT and p53-null mice were left untreated (control; upper panel), irradiated (13 Gy; middle panel), or irradiated and then given p53-WT BMT 24 h later (13 Gy + BMT; bottom panel). Mice were killed and femurs collected 5 days after IR and 1h after intraperitoneal injection of EdU (10 mg/kg mouse body weight). A diagram of a femur is shown in the upper left corner of the figure: dm-distal metaphysis, d-diaphysis, pm-proximal metaphysis. Representative results from three mice of each phenotype/treatment group are shown. Two boxed areas from each low magnification (×2.5) image are shown immediately below at higher magnification (×20).

The critical roles of hematopoietic cells in mediating inflammation and carcinogenesis associated with p53 deficiency have been established in numerous studies of the phenotypes of chimeric mice created by transplanting p53-deficient BM cells into irradiated p53-WT recipients^31–33^. We tried to model the opposite situation: to generate mice with p53-WT hematopoiesis on p53-null background. We tested different doses of TBI to find conditions suitable for the substitution of the hematopoietic system in these animals and comparing mouse survival. As expected^34^, p53-null mice irradiated with 13 Gy died earlier (4–6 days) than p53-WT mice (10–12 days) (GI syndrome) (Fig. 1A). However, although 100% of irradiated p53-WT mice were rescued by p53-WT BMT, p53-null mice died with essentially the same kinetics regardless of whether they received p53-WT BMT after irradiation or not (Fig. 1A). Consistent with our earlier results^34^, after 9 Gy TBI (HP syndrome), p53-null mice lived significantly longer than p53-WT mice (Fig. 1A). This difference in survival correlated with more rapid loss of white blood cells (lymphocytes, neutrophils, monocytes) in p53-WT mice compared with p53-null mice following TBI with lethal (9 Gy) and sublethal (6 Gy) doses of TBI (Fig. S1A). Immunofluorescent staining of BM tissue sections confirmed that the BM of p53-null mice contained more HP cells (CD45+) than the BM of p53-WT mice on the first several days after 6–15 Gy TBI (Fig. S1B). Despite the prolonged survival of p53-null mice compared with p53-WT mice after 9 Gy TBI, BMT with p53-WT BM cells failed to rescue these p53-null mice (Fig. 1A). Therefore, at both HP syndrome and GI syndrome-inducing IR dose levels, BMT was not sufficient to allow survival of p53-null hosts. This was the case for both males and females and regardless of whether p53-WT or p53-null BM was used for transplantation or transplantation was done 72 h after IR (Fig. S2A, B). A similar effect was observed when 6-TG treatment was used for preconditioning instead of TBI (Fig. S2D).

We compared the proliferation of BM cells, reflecting the intensity of hematopoiesis, in p53-null and p53-WT mice before and after TBI and BMT. EdU-positive cells were similarly and uniformly distributed in the BM of p53-WT and p53-null mice before irradiation (Fig. 1B; upper panel). TBI resulted in a dramatic reduction in EdU incorporation into BM cells of both mouse genotypes (Fig. 1B; middle panel). Numerous islands of EdU+ cells expressing lineage-specific markers (Fig. S2C) appeared in p53-WT mice but absent in BM of p53-null mice after TBI and BMT (Fig. 1B; lower panel).

### Transplanted BM cells can reach BM of p53-null mice but cannot proliferate there

**Fig. 2:**
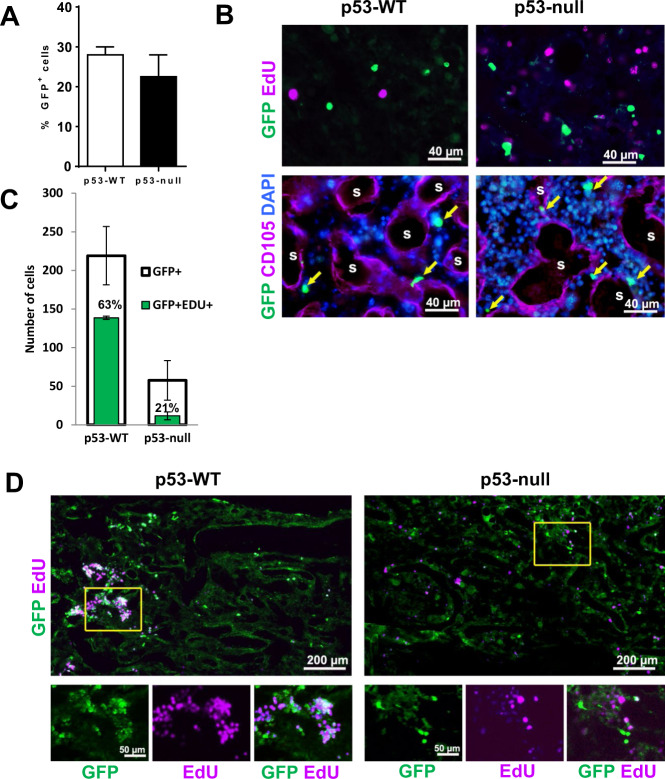
Transplanted BM cells migrate to the BM in p53-null mice, but do not proliferate there. **A**–**B** BM cells transplanted into irradiated mice by tail vein infusion migrate to the BM in both p53-WT and p53-null mice. **A** BM cells (1 × 10^7^) from GFP-expressing mice (C57BL/6-Tg(UBC-GFP) 30 Scha/J) were adoptively transferred via the tail vein into recipient syngeneic p53-WT or p53-null hosts (C57BL/6) that were preconditioned with 13 Gy TBI. In all, 24 h post BMT, BM cells were collected from the femurs of recipient mice to quantify GFP-expressing cells within the BM compartment by FACS (percentage of GFP+ cells among total BM cells). Results are mean from three mice analyzed individually. Error bars signify SEM. **B** Longitudinal sections of femoral BM from mice described in **A** were prepared 72 h post BMT and stained for EdU (proliferation marker), GFP (marker of donor cells), CD105 (marker of sinusoidal endothelium), and DAPI (DNA marker). “S”-sinusoid; yellow arrows indicate GFP+ cells. **C** Quantitation of the experiment described in **D**. The average number of GFP+ and EdU+GFP+ cells per the counted field is shown for p53-WT and p53-null BM 72 h post BMT (EdU+GFP+ and GFP+ cells were counted in three fields of the images taken with objective ×5). Error bars signify SEM. The difference between p53-null and p53-WT EdU+GFP+ cells was statistically significant (*P* = 0.009 by two-tailed *t* test). The percentage of Edu+GFP+ cells relative to all GFP+ cells is shown for p53-WT (63%) and p53-null (21%) mice. **D** Transplanted BM cells do not proliferate in the BM of p53-null mice. BM cells (1 × 10^7^) from GFP-expressing mice were adoptively transferred via the tail vein into recipient syngeneic p53-WT or p53-null hosts (C57BL/6) that were preconditioned with 11 Gy TBI. Longitudinal sections of femoral BM were prepared 72 h post BMT and stained for EdU and GFP. Boxed areas from each low magnification (×10) image are shown below at higher magnification (×40) with individual and overlapped stains. For **B** and **C**, EdU was injected intraperitoneally 1h before mouse sacrifice. Representative images from four mice of each genotype are shown and the area of the femur corresponding to the sections that are shown is indicated by a box on the schematic of the femur bone.

To explain the observed phenomenon. we considered two possibilities: (i) BM cells transplanted to p53-null mice do not home to the host BM, or (ii) transplanted BM cells reach the host BM but cannot proliferate there. To distinguish between these possibilities, we used GFP-expressing mice as a source of donor cells for BMT into p53-WT and p53-null mice that were preconditioned with 11 or 13 Gy TBI to see a consistency of results. There was no significant difference in the proportion of GFP+ cells in BM from p53-WT and p53-null mice 24 h after BMT (Fig. 2A, B) demonstrating that principal accessibility of BM by donor cells is similar between the two mouse genotypes. 24 h after BMT GFP+ cells in both genotypes were EdU-negative and located in the BM parenchyma between sinusoids (Fig. 2B). Three days after BMT, with both levels of TBI, we observed clusters of proliferating GFP+/EdU+ cells in p53-WT mice (Fig. 2D, left), but not in p53-null mice (Fig. 2D, C).

We concluded that transplanted BM cells reach the BM compartment similarly in p53-WT and p53-null mice, but they do not proliferate in the p53-deficient background.

### TBI induces αSMA-positive stromal cells in the BM of p53-WT but not in p53-null mice

**Fig. 3:**
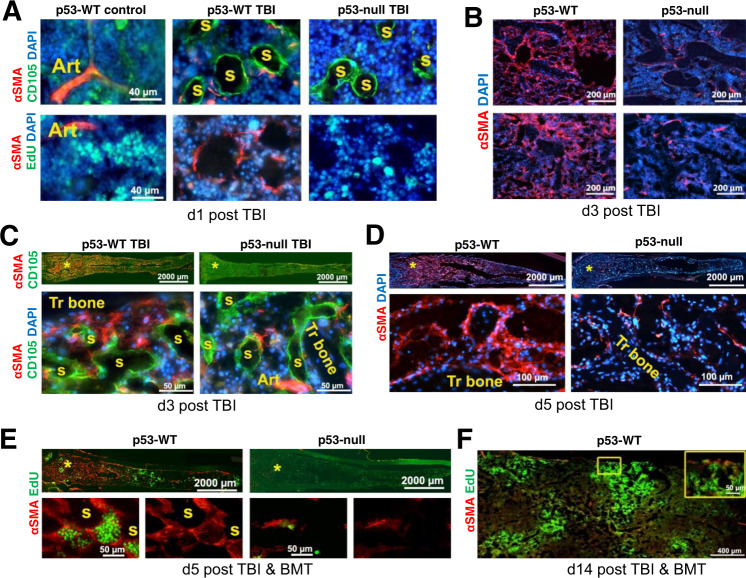
IR induces appearance of αSMA-positive fibroblast-like cells in the BM of p53-WT mice but not in the BM of p53-null mice. **A** Longitudinal sections of femoral BM (distal metaphysis area) from a control (non-irradiated) p53-WT mouse and irradiated (13 Gy) p53-WT and p53-null mice stained for αSMA, CD105, and DAPI (upper panel) or for αSMA, EdU, and DAPI (low panel). Analysis was performed 24 h post IR. EdU was injected intraperitoneally 1h before mouse sacrifice. **B** Massive appearance of αSMA cells in the BM of p53-WT mice was a result of irradiation and was not related to BMT. p53-WT and p53-null mice were irradiated with 6 Gy or 15 Gy of TBI (no BMT) and αSMA+ cells were detected in sections of femoral BM by immunofluorescence 3 days after irradiation. **C** Longitudinal sections of femoral BM from irradiated (13 Gy) p53-WT and p53-null mice prepared 3 days post IR. Upper panels: low magnification (×2.5) of whole femurs stained for αSMA and CD105. Lower panels: high magnification (×40) of boxed areas stained for αSMA, CD105, and DAPI. **D** Longitudinal sections of femoral BM from irradiated (13 Gy) p53-WT and p53-null mice prepared 5 days post IR and stained for αSMA and DAPI. Upper panels: low magnification (×2.5) of whole femurs; Lower panels: high magnification (×40) of boxed areas. **E** Longitudinal sections of femoral BM from IR p53-WT and p53-null mice after BMT (5 days after IR with 13 Gy) stained for αSMA and EdU. Upper panels: low magnification (×2.5) of whole femurs; lower panels: high magnification (×40) of boxed areas. **F** Longitudinal sections of femoral BM (distal metaphysis) from p53-WT mouse after BMT (14 days after BMT) were stained for αSMA and EdU. EdU was injected intraperitoneally 1h before mouse sacrifice. The inset panel shows the boxed area at high magnification (×40). For **A**–**F**: *S* sinusoid, *Art* arterial vessels, *Tr. Bone* trabecular bone. Stars in diagrams and in low imaging of femur show areas of fluorescent pictures. Representative images are shown from the analysis of 3–5 mice for each staining.

Recent works have identified perivascular cells as a major component of HSCs-niches^19,20^. One type of perivascular cells localized in arterial vessels of the BM expresses αSMA. αSMA+ stromal cells not associated with arterial vessels are commonly observed in fetal BM and in hematological diseases^35,36^. We proposed that this type of cells may be important for the successful engraftment of transplanted cells. To investigate this possibility, we compared changes in expression of αSMA in the BM of p53-WT and p53-null mice at different time points after TBI. In agreement with the literature^35^, control non-irradiated mice of both genotypes showed αSMA expression only in perivascular cells associated with BM arterial vessels (Fig. 3A, Fig. S3B). However, after TBI, αSMA+ cells were observed around sinusoids in the BM of p53-WT mice, but not in p53-null mice. The irradiation-induced αSMA+ cells seen in p53-WT BM were non-proliferative and had the morphology of stromal reticular cells with long processes (Fig. 3A, Fig. S3B).

The number of αSMA+ stromal cells was increased 3 and 5 days after TBI in p53-WT but not in p53-null BM (Fig. 3B–D). HP cell death, probably, was not a reason for activation of αSMA+ cells in p53-WT mice because at later time points (>5 days) it becomes similar in both genotypes. However, there was no accumulation of αSMA+ cells even at these later time points in p53-null mice. In the diaphysis, αSMA+ cells were concentrated predominately around the central venous sinus (Fig. S3A). Similar induction of αSMA+ cells in the BM of p53-WT, but not p53-null mice, were also observed with either lower or higher doses TBI (Fig. 3B). αSMA+ cells were host-derived since no cells positive for GFP and αSMA were observed in mice transplanted with BM from GFP-expressing mice (Fig. S3C). EdU labeling revealed islands of proliferating cells surrounded by αSMA+ cells in p53-WT (Fig. 3E, left), but not in p53-null (Fig. 3E, right) femurs after BMT suggesting that the αSMA cells are required for proliferation of transplanted BM cells. The presence of αSMA+ cells in the BM of p53-WT mice rescued from 13 Gy TBI by BMT declined substantially between Days 5 and 14 post BMT (Fig. 3E, F). However, large clusters of EdU+ cells remained visible, indicating ongoing intensive BM regeneration. Thus, the TBI-induced αSMA+ stromal cells are transiently present during the early steps of hematopoietic renewal and then disappear once intensive BM regeneration is underway.

Antibody staining showed that The TBI-induced αSMA+ cells were negative for the hematopoietic marker CD45, endothelial markers Mega-32^18,37^, CD105^38^, and CD146^39^, the macrophage marker F4/80^40^, the mature adipocyte marker perilipin, fibroblast markers collagen type-I, fibroblast-specific protein 1 (S100A4), and CXCL12, perivascular stromal cells marker LepR^14^. Some αSMA+ cells were positive for NG2+, marker of arteriolar pericytes (Fig. S3D).

Transient aSMA+ cells were also detected in the areas of intensive vessel growth in BM of femurs of 3 week-old mice regardless of p53 genotype in the absence of irradiation (Fig. S4A).

### Differential induction of adiposity by TBI in the BM of p53-WT and p53-null mice

**Fig. 4:**
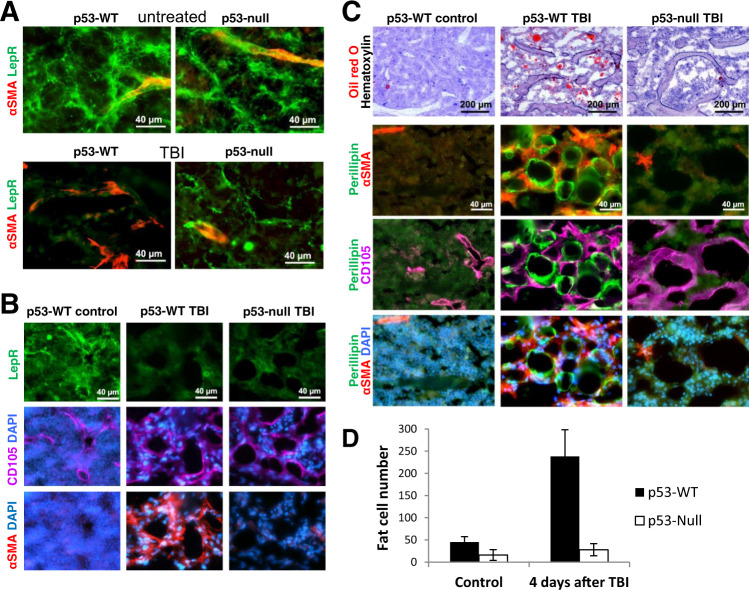
IR induces adipocyte differentiation in the BM of p53-WT mice but not p53-null mice. **A** Longitudinal sections of femoral BM (distal metaphysis) were prepared from control (non-irradiated) and irradiated (13 Gy) p53-WT and p53-null mice 24 h after IR and stained for LepR and αSMA expression. **B** Longitudinal sections of femoral BM (distal metaphysis) were prepared from control (non-irradiated) p53-WT mouse and from irradiated (11 Gy) p53-WT and p53-null mice 4 days after IR and stained for LepR, αSMA, and CD105 expression. DAPI was used as a costain to visualize nuclei. **C** Longitudinal sections of femoral BM (distal metaphysis) as described in **B** were stained with Oil Red O and hematoxylin (upper panel) and for perilipin, CD105, αSMA, and DAPI (lower panels). **D** Quantitation for Oil Red O-positive cells (in **C**, upper panels) in p53-WT and p53-null BM before and after TBI. Oil Red O-positive cells were counted from the images (five fields) taken with objective ×5. Error bars signify SEM. The difference between the number p53-null and p53-WT fat cells per counted field was statistically significant (*P* = 0.005 by two-tailed *t* test).

LepR+ perisinusoidal stromal cells, a multipotent subpopulation that can differentiate into adipocytes, osteoblasts, and chondrocytes (losing LepR expression)^29^ were recently shown to be an important component of BM HSC/HPC niches^14^. BM regeneration after IR damage is accompanied by increased adiposity within the BM^29^. Although the functional significance of this change remains unclear, we assessed the effect of TBI on the LepR+ stromal cells in the BM of p53-WT and p53-null mice. Before TBI, LepR+ cells were observed in the BM of both p53-WT and p53-null mice (Fig. 4A; Fig. S4B). One day after TBI, LepR expression disappeared, more quickly in p53-WT mice compared with p53-null mice, in the area of the massive appearance of αSMA+ cells (Fig. 4A, B). Four days after TBI, LepR+ cells were completely substituted by αSMA+ cells in p53-WT, but not in p53-null mice (Fig. 4B).

Oil Red O staining showed an increase in the number of adipocytes on the fourth day after TBI in the BM of p53-WT, but not in p53-null mice with any time and dose tested (Fig. 4C, D, Fig. S4C). Another adipocyte marker (perilipin) gave similar results (Fig. 4C). BM-transplanted p53-WT mice showed the presence of numerous adipocytes within the regenerating BM on Day 11 post BMT (Fig. S4D) that correlated with the disappearance of αSMA+ cells from the BM (Fig. S4D). Thus, in p53-null BM, the absence of TBI-induced αSMA+ cells correlated with decreased adiposity.

### Differential damage of sinusoidal system in BM of p53-null and p53-WT mice

**Fig. 5:**
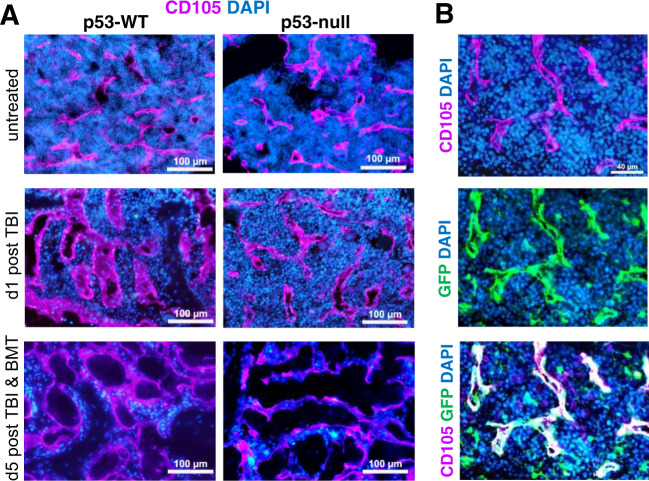
Sinusoidal system in BM of p53-null mice is more damaged after IR and BMT compared with p53-WT mice. **A** Dynamics of BM sinusoidal system re-arrangement in p53-WT and p53-null mice after IR without or with BMT. Femoral BM sections were prepared at the indicated times from 53-WT and p53-null mice that were irradiated (13 Gy TBI) without or with subsequent BMT (5 × 10^6^ cells from p53-WT mice 24 h post irradiation). Sections were stained for CD105 (magenta), a marker of sinusoid endothelium, and with DAPI (blue). Representative images from 2–3 mice of each genotype are shown. **B** The regenerated endothelium in irradiated p53-WT mice after BMT is exclusive of host origin. p53-WT GFP-expressing transgenic mice were irradiated (13 Gy) and then transplanted with syngeneic GFP-negative BM cells 24 h post IR. Immunofluorescence was performed on BM sections collected 6 months after IR/BMT. Green–GFP, Blue–DAPI, Magenta–CD105 (sinusoidal endothelium marker). Representative images from two mice are shown.

The degree of TBI injury of the stromal cells including the sinusoidal system dependent on the irradiation dose^41^ determines the success of BMT. We assessed a difference in the extent of TBI-induced injury to the BM sinusoidal system in p53-WT versus p53-null mice. One day after TBI (without BMT), sinusoids were noticeably less dilated in p53-null mice than in p53-WT mice (Fig. 5A), probably, due to the higher hematopoietic cellularity supporting sinusoids^42^. On the fifth day after TBI (without BMT), sinusoids in the BM of p53-WT and p53-null mice displayed similar levels of IR-induced damage. When TBI was followed by BMT, the BM vascular system in p53-WT mice appeared less damaged than in mice without BMT (Fig. 5A). p53-null mice had substantial BM vasculature damage even after BMT (Fig. 5A). Analysis of BM from lethally irradiated GFP+/p53-WT mice that were rescued by BMT from p53-WT non-GFP-expressing donor mice showed regeneration of the BM sinusoidal system from host cells at 6 months post-TBI/BMT (Fig. 5B).

These results indicate that the BM sinusoidal system is more susceptible to TBI-induced damage in p53-null mice and that its regeneration after BMT requires p53.

### Differential radiosensitivity of BAMC from p53-null and p53-WT mice

We characterized and compared rediosensitivity in vitro of another BM stromal constituent, bone-adherent mesenchymal cells (BAMC) from p53-WT and p53-null mice. Both cell populations were similar in the expression of fibroblast markers (collagen type-I and vimentin) and pericyte markers (αSMA and NG2) (Fig. S5A). The majority of BAMC of both genotypes expressed αSMA in vitro without irradiation (Fig. S5A). Upon adipogenic stimulation, there was a pronounced adipocyte differentiation in p53-WT BAMC cultures but not in p53-null cultures (Fig. S5B).

**Fig. 6:**
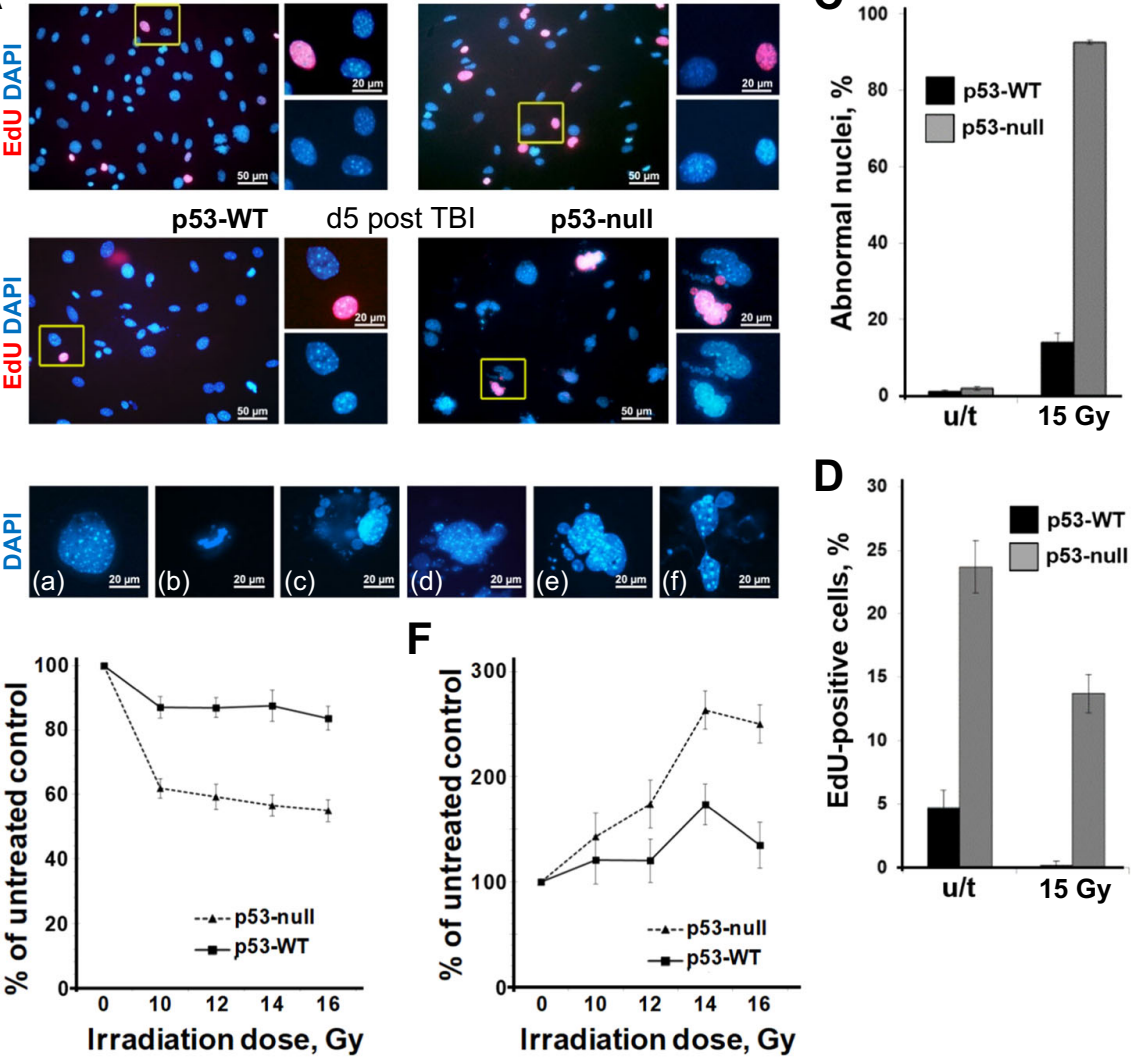
Bone-adherent mesenchymal cells (BAMC) from p53-null mice are more radiosensitive than those from p53-WT mice. **A** A number of nuclei with abnormal morphology are higher in p53-null BAMC than in p53-WT BAMC after IR. Cultures of BAMC from p53-WT and p53-null mice were evaluated by immunofluorescence before IR (upper panel) or 5 days after in vitro IR (15 Gy) (bottom panel). Cells were stained to detect EdU incorporation and DAPI as a counterstain to visualize nuclei. EdU was added to cell cultures 1h before staining. Data from **A** (quantitation) are shown graphically in **C** and **D**. **B** Images of individual cells provide examples of abnormal nuclear morphology observed in irradiated p53-null BAMC cultures (15 Gy, 5 days). Nuclei in p53-null cells had different morphological types of abnormalities including (a) abnormally large nuclei, (b) condensed nuclear chromatin, (c) micronuclei, (d) nuclear budding, (e) large multilobed nuclei, (f) chromatin bridge. **C**, **D** Percent of EdU+ nuclei and abnormal nuclei relative to total cells was determined by counting cells under microscope in randomly selected areas (at least 500 nuclei of each sample). (Mean ± SD for three replicates from one BAMC culture). The difference in % of EdU between p53-KO and p53-WT BAMC was statistically significant (*p* < 0.003 for cells before irradiation, and *p* < 0.006 for cells after irradiation two-sided *t* test). The difference in % of abnormal nuclei between p53-KO and p53-WT BAMC was statistically not significant before IR, and significant after IR (*p* < 0. 0003 by two-sided *t* test). **E**, **F** BAMC were isolated from p53-null and p53-WT mouse femurs, expanded in vitro (three passages), and irradiated with different doses of IR (0–16 Gy). Cell viability was determined 72 h after IR by ATP assay (**E**) or by protease activity assay (CellTox Green assay) (**F**) and is shown relative to viability of untreated (non-irradiated) control cultures (mean ± SD for three replicates from one BAMC culture). The difference in viability between p53-KO and p53-WT BAMC was statistically significant (*p* < 0.05, two-sided *t* test) for all IR doses by ATP assay and for doses > 12 Gy by CellTox Green assay.

A higher percent of EdU+ cells was maintained in p53-null BAMC before (24%) and after (14%) 15 Gy of IR (5 days) (Fig. 6A, D) compared with p53-WT mice (5% and 1%). More than 90% of p53-null cells had abnormal nuclei morphology (Fig. 6B (a–f)) (including typical morphology of mitotic catastrophe) compared with 14% of 53-WT cells 5 and 7 days after irradiation (Fig. 6B, C; Fig. S6A, B).

p53-null BAMC were significantly more sensitive to irradiation than p53-WT BAMC, particularly at higher irradiation doses, determined by cell viability assays (Fig. 6E, F). Taken together, our results clearly indicate that p53 deficiency results in greater radiosensitivity of BM stromal cells.

We compared the distribution of p53-WT and p53-null BAMC cells among the phases of the cell cycle – before and after irradiation. The proportion of p53-WT cells in S-phase dropped down to nearly zero already 24 h post irradiation, in contrast to p53-null cells, which continued to proceed through the cycle (Fig. S7A). This is consistent with the proportion of cells incorporating EdU on day 5 post irradiation: in p53-WT cells EdU+ cells were undetectable, whereas p53-null cells continued to replicate their DNA (Fig. 6D).

Global gene expression profiling was done with RNA samples from freshly isolated BAMC of intact p53-WT and p53-null mice (control) and 24 h after 13 Gy TBI (GEO repository, accession number GSE117625). Transcriptomes of intact BAMC of both genotypes were closely similar. TBI causes strong changes in transcriptomes of p53-null BAMC as compared with minor changes in the transcriptome of wild-type BAMC (Fig. S8). Massive drop in expression of numerous genes in irradiated p53-null BAMC is consistent with high sensitivity of these cells to radiation and can be explained by their massive loss. Therefore, we did not analyze individual genes that differentially expressed between BAMC from p53-WT and p53-null mice.

### Supplementation of BM with BAMC increases survival of p53-null mice after TBI and BMT

**Fig. 7:**
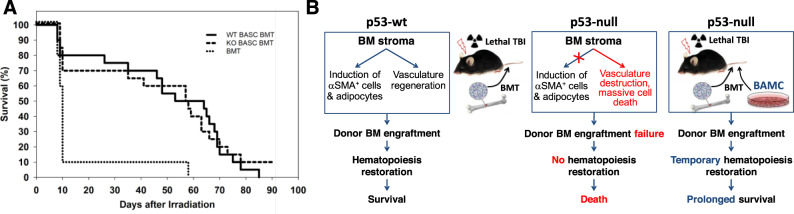
Supplementation of BM with BAMC improves survival of p53-null mice after IR and BMT. **A** Three groups of p53-null mice (*n* = 20/group) received total BM cells (5 × 10^6^) from p53-WT mice by tail vein infusion 48h after 12 Gy TBI. Two groups also received 3 × 10^5^ BAMC from p53-WT or p53-null mice, respectively, at the same time via tail vein. Survival of recipient mice was followed for 90 days after IR. The difference in survival fraction (%) between mice given BMT only and mice given BMT plus BAMC from p53-WT or p53-null mice was statistically significant (*p* < 0.01 by two-tailed Fisher’s exact test) up to day 60 post IR, at which point all BMT only mice were dead. **B** Scheme: p53 determines radiosensitivity of hematopoietic and radioresistance of stromal component of BM. p53-null mice cannot be rescued by BMT from any lethal dose of total body radiation, whereas this procedure enables survival of p53 wild-type mice. Hematopoietic recovery after IR and BMT in p53-WT mice correlated with the appearance IR-induced αSMA+ stromal cell population and increased adiposity which were absent in p53-null mice. The level of IR-induced injury of BM stromal compartment in p53-null mice is higher compared with p53-WT mice and cannot support the proliferation of hematopoietic cells after BM engraftment. Supplementation of the donor BM with bone marrow adherent stromal cells from non-irradiated mice enables engraftment of pluripotent hematopoietic precursors and temporary restoration of hematopoiesis in irradiated p53-null mice.

The data described above suggest that BMT fails to rescue lethally irradiated p53-null mice because an adequate BM stromal environment is not maintained in the recipient mice. To directly test whether the response of p53-null stroma to TBI determines the failure of subsequent BMT, we evaluated the effect of transplanted p53-WT or p53-null BAMC on survival of p53-null mice after TBI with a dose 12 Gy because p53-null mice quickly died from this dose and could not be rescued by BMT. Moreover, this dose is still in the range of HP syndrome-induced death. Three groups of p53-null mice received TBI (12 Gy) and p53-WT BM cells (5 × 10^6^) 48h post-TBI. At the same time, two of these groups also received 3 × 10^5^ BAMC from either p53-WT or p53-null mice. Transplantation of BAMC of both p53 genotypes significantly prolong survival p53-null mice after lethal TBI with BMT (Fig. 7). By day 10, 90% of mice received only BMT were dead, whereas most of mice obtained p53-null (80%) or p53-WT (70%) BAMC were alive. It is noteworthy that the inclusion of intact BAMC in donor BM provide only temporary support for donor hematopoiesis (up to 100 days max) presumably due to insufficient restoration of the stromal niche by supplementing BM with mesenchymal cells alone.

To confirm that BAMC could reach BM of recipient mice, we co-transplanted 3 × 10^5^ BAMC from p53-WT GFP+ isogenic mice with 5 × 10^6^ p53-WT BM cells to irradiated (13 Gy) p53-WT and p53-null mice. Six days after BMT, we detected GFP+ BAMC in BM of mice of both genotypes (Fig. S7B).

## Discussion

In this study, we found that the success of BMT following IR preconditioning is determined by p53. Unlike p53-WT mice, p53-null mice irradiated with lethal doses of TBI could not be rescued by adoptive transfer of BM cells from either p53-WT or p53-null mice and died with kinetics similar to mice irradiated without BMT. The failure of BMT on the p53-null background was due to failure of the transplanted donor cells to proliferate after reaching the BM. Although we did not test a probability of poor engraftment due to the delay of the p53-/- hematopoietic cell death, we think that it is low. Thus, p53 function is required for supporting the viability and proliferation of donor cells.

Successful regeneration of BM from donor cells in irradiated p53-WT mice correlated with the massive appearance of αSMA+ stromal cells. This suggests that after genotoxic stress, the differentiation program directing conversion of precursor cells into αSMA+ cells might be p53-dependent. Some earlier data showed regulation of αSMA expression by p53 under non-stressed conditions in certain cell types, but not in mesenchymal cells^43,44^. In our work, the difference in αSMA expression in p53-WT versus p53-null mice was evident only after TBI, in basal conditions αSMA expression was not p53-dependent. Based on their fibroblast-like morphology, perivascular location, and expression of αSMA, TBI-induced αSMA+ cells could be considered stromal mesenchymal cells. However, these cells did not express known markers for a variety of different BM stromal cell populations. A subset of αSMA-expressing macrophages can maintain HSC/HPC and protect them during alarm situations^45^. We found that αSMA+ stromal cells do not incorporate EdU in irradiated p53-WT mice which is consistent with data showing that expression of the alpha isoform of SMA correlated with growth arrest of fibroblasts^43,46,47^. Since αSMA+ cells were observed to be in tight association with endothelial cells of damaged BM sinuses, we propose that they support their restoration and could be niche-regulating stromal cells responsible for hematopoietic recovery after IR and BMT.

Previous studies have shown that BM regeneration after radiation damage is associated with increased adiposity^29^ of the BM and that BM adipose tissue may produce factors that affect hematopoiesis^48,49^. Our data indicate that the pathway directing TBI-induced adipocyte differentiation requires p53 function. Probably, αSMA+ cells that appear only in irradiated p53-WT mice originate from a cluster of adipo-primed cells found after 5-FU treatment of Lepr+ cells^28^. Boregowda et al. also found that p53 in MSCs is needed to promote adipocytic differentiation^50^.

Although BM stromal cells are clearly less radiosensitive than hematopoietic cells, they can be damaged by high doses of radiation. Dramatically reduced clonogenic capacity of BM mesenchymal progenitors was found in patients after BMT^51–55^. Co-transplantation of MSCs with HSCs improved donor engraftment, hematopoietic recovery, and survival of mice^9,10,24,56,57^. Interestingly, complete restoration of the clonogenic capacity of BM stroma after BMT occurs only in younger than 5 years old patients^52,54^. This might be explained by the greater presence of αSMA+ cells in their BM as in young animals.

The mechanism of the radioprotective role of p53 in BM stroma can involve p53-mediated suppression of mitotic catastrophe, as shown in the present and earlier works for small intestine and tumors^34,58–60^. Other mechanisms may elevate radiosensitivity of p53-null stromal cells including activation of apoptosis by desilencing of endogenous retroelements^61^. p53 is also known to protect cells from oxidative stress^62^ and activate DNA repair mechanisms after DNA damage^63^. p53 regulation of *Notch* may contribute to the recovery of BM endothelium following irradiation^64^. Finally, p53 might regulate the transition of fibroblast-like cells to endothelial cells to restore them after injury^65^.

The fact that p53 might act as a survival factor in stromal cells could be exploited in cancer therapy. As tumor stromal cells are not part of the tumor per se, but are originated from the host, they should be wild-type for p53. Therefore, targeting p53 in tumor stroma is a plausible approach for improving the efficacy of anticancer radio- and chemotherapy in cases where the tumors would not benefit from p53 suppression. In fact, our earlier data demonstrated that both genetic and pharmacologic suppression of p53 in tumor stroma strongly sensitizes p53-deficient tumors to radio- and chemotherapy^58^.

In summary, the importance of p53 for the success of BMT after TBI preconditioning is twofold, with it mediating apoptotic death of hematopoietic cells while regulating survival and possibly appropriate differentiation of BM stromal cells (scheme in Fig. 7B).

## Materials and methods

### Animals

p53-null mice on a C57BL/6 background and C57BL/6-Tg(UBC-GFP) 30Scha/J mice were obtained from Jackson Laboratories (Bar Harbor, ME) and then maintained as colonies in the Roswell Park Cancer Institute (RPCI) animal facility. All animal studies were conducted in accordance with the recommendations in the Guide for the Care and Use of Laboratory Animals of the Association for the Assessment and Accreditation of Laboratory Animal Care International (AAALAC). The experiment protocol was approved by the Institutional Animal Care and Use Committee (IACUC) at the RPCI (protocol # 1081). Mice were assigned randomly to groups; group sizes were selected based on prior experience. No animals were excluded from further analysis in the reported studies.

### Isolation of BAMC and in vitro cell viability assays

BAMC were isolated and purified from 10–12 weeks-old WT and p53-null mice as described^66^. Passaging was performed by replating the cells at 5 × 10^4^ cells/cm^2^ in αMEM medium (Sigma M4526). BAMC differentiation into adipocytes was tested using an adipogenic supplement from the Mouse Mesenchymal Stem Cell Functional Identification Kit (R&D Systems, Minneapolis, MN; Cat.#SC010) according to the manufacturer’s instructions. The viability of cells was determined using ATP Cell Viability Assay, and CellTox Green Assay kits (Promega, Madison, WI).

### Irradiation of mice and cells, 6-Thioguanine (6-TG) treatment

BAMS cells were plated in 96-well plates (Corning Costar, Tewksbury, MA) at a density 10,000 cells/well. Mice (TBI) and cells were irradiated using a ^137^Cs Mark I-30 irradiator (J. L. Shepherd and Associates) with a dose rate of 2.2 Gy/min. Mice were subcutaneously injected (5 mg/mouse per day, 7 days) with 0.2 ml 6-TG solution (Sigma Chemical Co., St. Louis, MO).

BMT was performed as described previously^34^. In brief, 5 × 10^6^ cells/mouse were delivered via tail vein 24 h, 48h, or 72 h after irradiation.

### Immunofluorescent staining

Immunostaining of BM was performed on cryosections of paraformaldehyde-fixed (4%) and decalcified (0.5 M ethylenediaminetetraacetic acid, 7 days) mouse femurs embedded in Neg-50 cryo-medium (Fisher Scientific, Hampton, NH). Images were acquired with Zeiss Axio Imager Z1 fluorescence microscope using AxioCam MRc & MRm-Fl (Carl Zeiss, Jena, Germany) and their evaluation was blindly performed. Used antibodies are listed in Supplemental Table S1 and S2.

### EdU proliferation assay

Mice were injected with EdU (10 mg/kg in PBS) intraperitoneally 1 h prior to tissue collection. Cells were treated with 10 μg/ml for 1h before fixation in 4% formaldehyde. Click-iT Plus EdU Imaging Kit (Invitrogen, Grand Island, NY) with AlexaFluor 488 (Cat.#C10637) or 647 (Cat.#C10639) was used according to the manufacturer’s recommendations. Slides were mounted in ProLong™ gold antifade medium with DAPI (ThermoFisher Scientific, Cat.#p36931). Percentage of cells with EdU+ nuclei and abnormal nuclei were determined by counting under the microscope in randomly selected three areas (at least 200 cells in each area).

### Quantification of GFP-positive cells by FACS

BM cells (1 × 10^7^) from GFP-expressing mice (C57BL/6-Tg(UBC-GFP) 30Scha/J) were transferred via the tail vein into recipient hosts 24 h after their irradiation (13 Gy). 24 h after transfer, BM cells were collected from the femurs of recipient mice to quantify GFP-expressing cells by fluorescence-activated cell sorting (FACS). The percentage of GFP+ cells among total BM cells was determined excluding erythrocytes. Data were acquired on an LSRII Fortessa FACScan instrument (Becton Dickinson) and analyzed using WinList software (Verity House Software).

### Cell cycle analysis

BAMC were isolated from femurs of 6-week-old WT and p53-null mice (*n* = 10) as described^66^. Growing cells were irradiated with 10 Gy (second passage). In all, 24 h later cells were fixed in 70% ethanol at 4°C overnight. Cells were incubated in PBS with 50 μg/ml PI (BioLegend Inc., San Diego, CA, USA) and 100 μg RNase A (Invitrogen, Carlsbad, CA, USA). Data were acquired on an LSRII Fortessa FACScan flow cytometer (Becton Dickinson), stored in Listmode format, and analyzed using ModFit 4.0 software (Verity Software House). Scatter of the cells and doublet discrimination were criteria used prior to analyzing the DNA content of PI-stained cells.

### Microarray analysis of transcriptomes

Gene expression profiling was done by the RPCI Genomics Shared Facility using Mouse WG-6 whole-genome gene expression assay and direct hybridization assay (Illumina, San Diego, CA, USA). RNA was prepared from BAMCs isolated from p53-WT and p53-null mice (three mice/group) either unirradiated or 24 h after 13 Gy of irradiation. Quantile normalization and background subtraction was conducted using Illumina Genestudio.

### Statistical analysis

One representative experimental data set is shown from two or three independent experiments. Differences between groups within experiments were analyzed using two-tailed unpaired Student’s *t* test and Fisher’s exact test. Animal survival Kaplan–Meier curves were compared using the log-rank test. *P* < 0.05 was considered statistically significant.

## Supplementary information

Supplemenatl material
