# Supplementary material for: Resistance of bone marrow stroma to genotoxic preconditioning is determined by p53
Source: Cell Death Dis. 2021 May 26;12(6):545. doi: 10.1038/s41419-021-03824-3 (PMC8154997; doi:10.1038/s41419-021-03824-3)
Supplement: Supplementary file 1 — Supplemenatl material [file 41419_2021_3824_MOESM1_ESM.pdf]

Supplementary Information for:

Radiation resistance of bone marrow stroma that enables donor bone marrow engraftment in mice is determined by p53

Natalia Fedtsova[a,1], Elena A. Komarova[a,1], Kellee F. Greene[a], Liliya R. Novototskaya[a], Ivan Molodtsov[b], Craig M. Brackett[a], Evguenia Strom[c], Anatoli S. Gleiberman[c], Alexander N. Shakhov[d], and Andrei V. Gudkov[a,c,2]

Correspondence author: A.V. Gudkov, Department of Cell Stress Biology, Roswell Park Cancer Institute, Buffalo 14263, NY, USA; Tel: +1 716 8453902; Fax: +1 716 8453944;

E-mail: andrei.gudkov@roswellpark.org

This PDF file includes:

Figs. S1 to S8
Tables S1 to S2

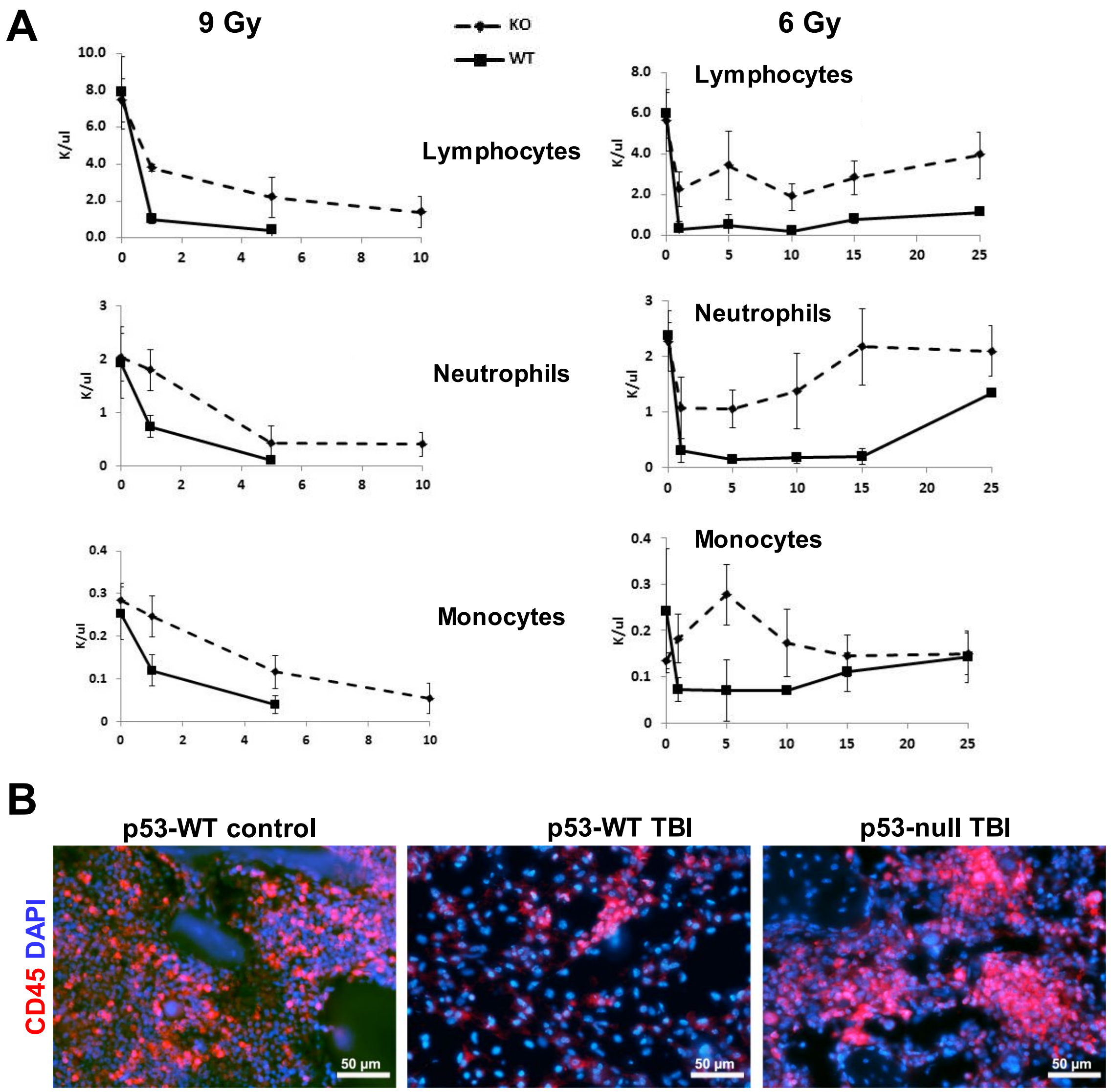

Fig. S1. Irradiation has a stronger negative impact on the hematopoietic system in p53-WT mice than in p53-null mice. (A) Levels of white blood cells (neutrophils, lymphocytes, and monocytes) drop more rapidly in p53-WT mice than in p53-null mice after IR. Groups of p53-KO (n=10) and p53-WT (n=8) mice were irradiated with 9 Gy or 6 Gy of IR. Levels of different types of white blood cells were measured in blood samples collected on the indicated days after IR. Differences between p53-KO and p53-WT groups after 6 Gy IR were statistically significant after 6 Gy IR on days 1 to 25 for neutrophils (Neu) and lymphocytes (Ly) and on days 1 to 10 for monocytes (Mo) and differences after 9 Gy IR were statistically significant on days 1 to 5 for Neu, Ly and Mo ($P < 0.05$ by two-tailed t-test). (B) The BM of p53-null mice contained more CD45+ hematopoietic cells than the BM of p53-WT mice after IR. Immunofluorescent staining for CD45 expression in longitudinal sections of femoral BM at the level of distal metaphysis from control (non-irradiated) p53-WT mice and irradiated (15 Gy TBI) p53-WT and p53-null mice. Analysis was performed three days after TBI with DAPI as a costain to visualize nuclei. Representative results from analysis of 3 mice per group are shown.

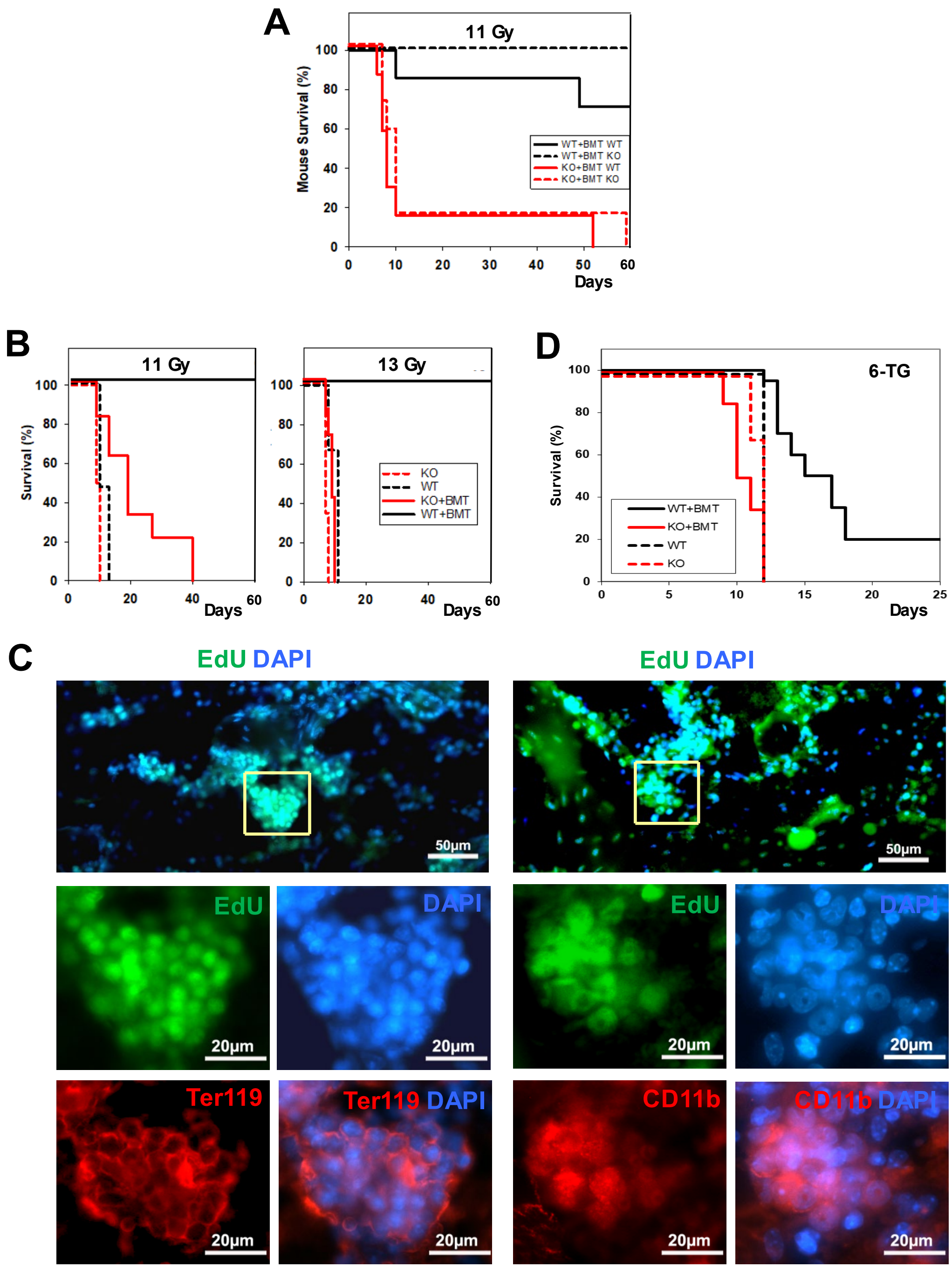

Supplemental Figure S2. BMT fails to rescue lethally irradiated p53-null regardless of whether the transplanted BM cells express p53 or not. (*A*) Groups of p53-KO and p53-WT mice (7 mice/group) were irradiated (11 Gy TBI) and given BMT with p53-KO or p53-WT BM cells ($5x10^6$/mouse) 24h later. Survival of mice (%) was followed for 60 days after IR. The difference in survival for mice of a given genotype transplanted with p53-WT or p53-KO BM was statistically not significant at all days (two-tailed Fisher's exact test). The difference in survival between mice of different genotypes transplanted with p53-WT BM or between mice of different genotypes transplanted with p53-KO BM was statistically significant ($P<0.03$ by two-tailed Fisher's exact test) beginning 10 days after IR. (*B*) Transplantation of BM 72h after IR did not change the survival of p53-null mice. p53-KO and P53-WT groups of mice (7-10 mice in each group) were irradiated with 11 or 13 Gy of IR. The followed BMT of P53-WT BM cells ($5x10^6$) was done 72h after IR (instead of 24h). Survival of mice (%) was followed for 120 days after IR. The difference in survival

between p53-WT and p53-KO mice and transplanted with p53-WT BM was statistically significant ($P<0.003$ beginning 19 days till 120 days for mice with 11 Gy of TBI and P< 0,00001 beginning 10 days till 120 days for mice with 13 Gy of TBI by two-tailed Fisher's exact test) (*C*) 5 days after TBI (13 Gy) and BMT, clusters of EdU+ proliferating cells are present in the BM of p53-WT mice but not in the BM of p53-null mice (Fig.1B)**.** EdU+ cell clusters represent colonies of different hematopoietic lineages as judged by their differential immunofluorescent staining for lineage-specific markers. As examples, colonies positive for erythroid (Ter119) and myeloid (CD11b) markers are shown. Cells in CD11b+ colonies had segmented nuclei (DAPI staining) specific for differentiated neutrophils. *(D)* p53-KO and p53-WT mice (n=20 in each group) were injected 7 times daily with 5mg/mouse of 6-thioguanine (6-TG) followed by p53-WT BMT ($5x10^6$ cells). Control groups (n=5) groups received similar treatment 6-TG with no BMT.

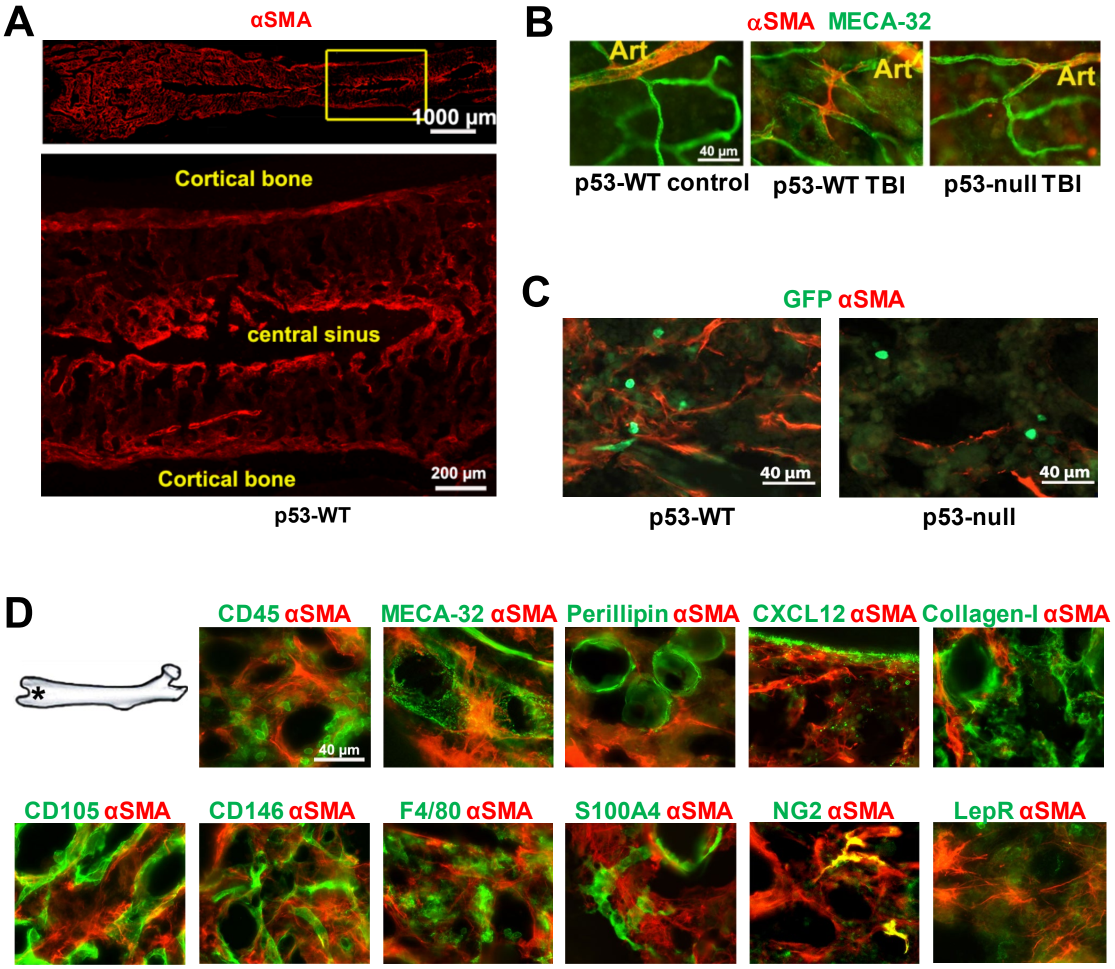

Fig. S3. (A) αSMA+ cells are induced by IR to a greater degree in p53-WT mice than p53-null mice. In the diaphysis region of the femoral BM, αSMA+ cells were concentrated predominately around the central venous sinus and close to cortical bone. Longitudinal sections of whole femurs from p53-WT mice 5 days after 13 Gy of IR stained for αSMA. Boxes showed the area used for high magnification. (B) Whole mount staining of BM from the diaphysis region of the femur from a control (non-irradiated) p53-WT mouse and irradiated (11 Gy) p53-WT and p53-null mice for αSMA and MECA-32. Analysis was performed 48h post-TBI. (C) Transplantation of BM from GFP-mice to lethally irradiated p53-WT and p53-null mice shows that donor cells do not transform into αSMA+ cells. p53-WT and p53-null mice were irradiated (13 Gy TBI) and given BMT with BM cells from p53-WT, GFP-expressing mice 24h later. αSMA and GFP expression in sections of femoral BM (distal metaphysis) prepared 3 days post-BMT was evaluated by immunofluorescence. (D) Expression of αSMA and known markers of distinct BM cell populations in the BM of irradiated p53-WT mice. Immunofluorescent staining for the indicated markers was performed on femoral BM sections from p53-WT mice 3 days after irradiation (15 Gy TBI). Stars in diagrams of femur marked areas of fluorescent images below.

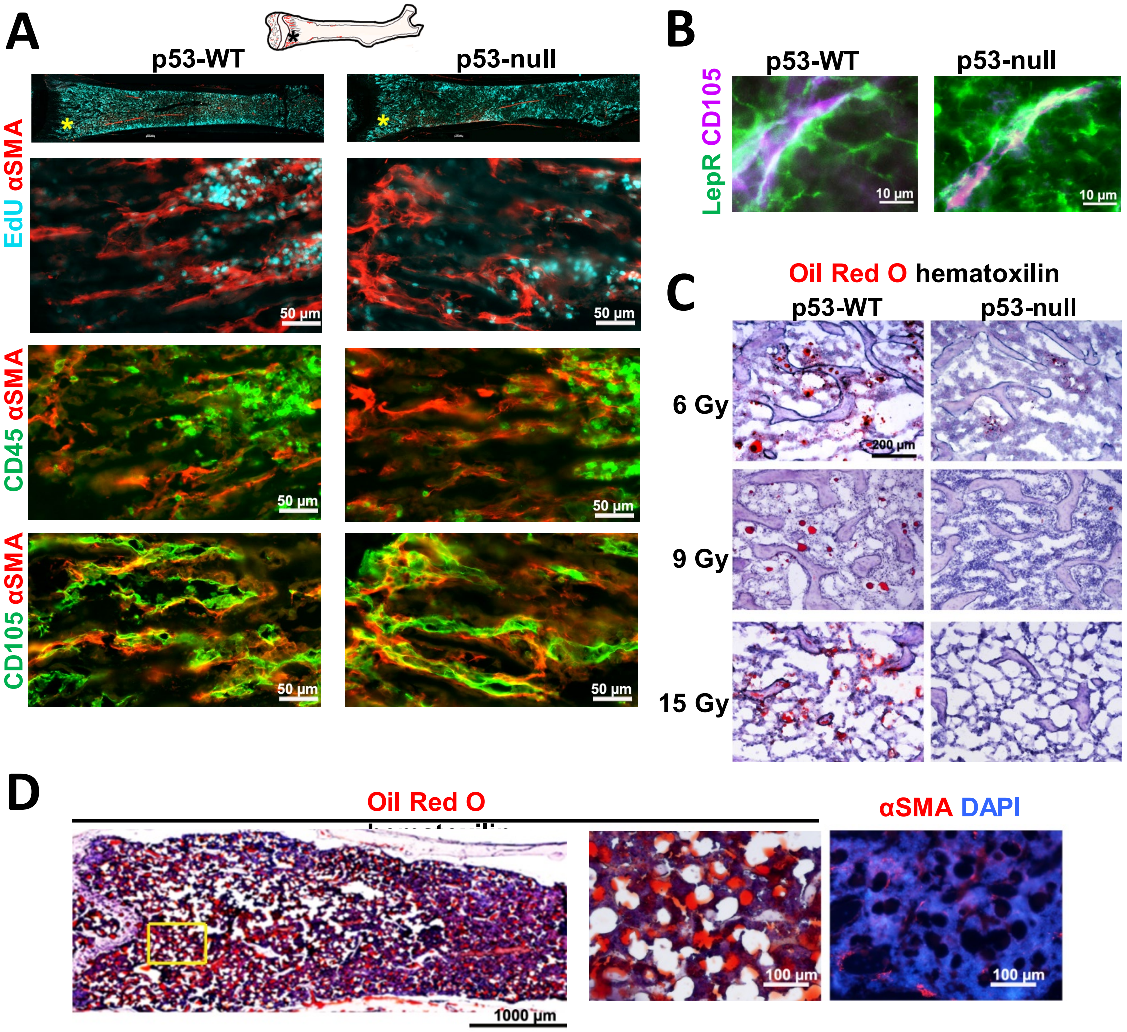

Fig. S4. (A) BM stromal cells of non-irradiated 3-week-old mice located close to CD105+ endothelial cells in areas of intensive vessel growth express a-SMA in both p53-WT and p53-null mice. Localization of αSMA+ cells (red) is shown on the scheme of femur. The longitudinal sections of the femoral BM at the level of distal metaphysis from the p53-WT and p53-null mice were double stained for αSMA and CD105, CD45 or EdU. Stars in diagrams of femur marked areas of fluorescent images below. (B) LepR+ cells localize around sinusoids (CD105+) in non-irradiated p53-WT and p53-null mice. Femoral BM sections from a control (non-irradiated) p53-WT mouse were stained to detect LepR and CD105 expression. (C) IR induced an increase in adiposity in the BM of p53-WT mice, but not p53-null mice. p53-WT and p53-null mice were irradiated (6 Gy, 13 Gy, or 15 Gy TBI) with no subsequent BMT. Femoral BM sections prepared 3 days (6 Gy and 15 Gy) or 7 days (13 Gy) after IR were stained with hematoxylin andOil Red O staining to detect adipocytes. Images from the distal metaphysis region of the BM are shown. Stars in diagrams of femur marked areas of images below. (D) In contrast to p53-null mice which do not survive after IR and BMT, p53-WT mice displayed BM regeneration accompanied by the appearance of numerous fat cells and coincident loss of αSMA+ cells. p53-WT mice were irradiated (13 Gy) and given BMT 24 h later with BM cells from a non-irradiated p53-WT donor mouse. Femoral BM sections prepared 14 days after BMT were stained with hematoxylin and Oil Red O to detect adipocytes (boxed area of image is shown at higher magnification below). Parallel section is stained for αSMA and DAPI.

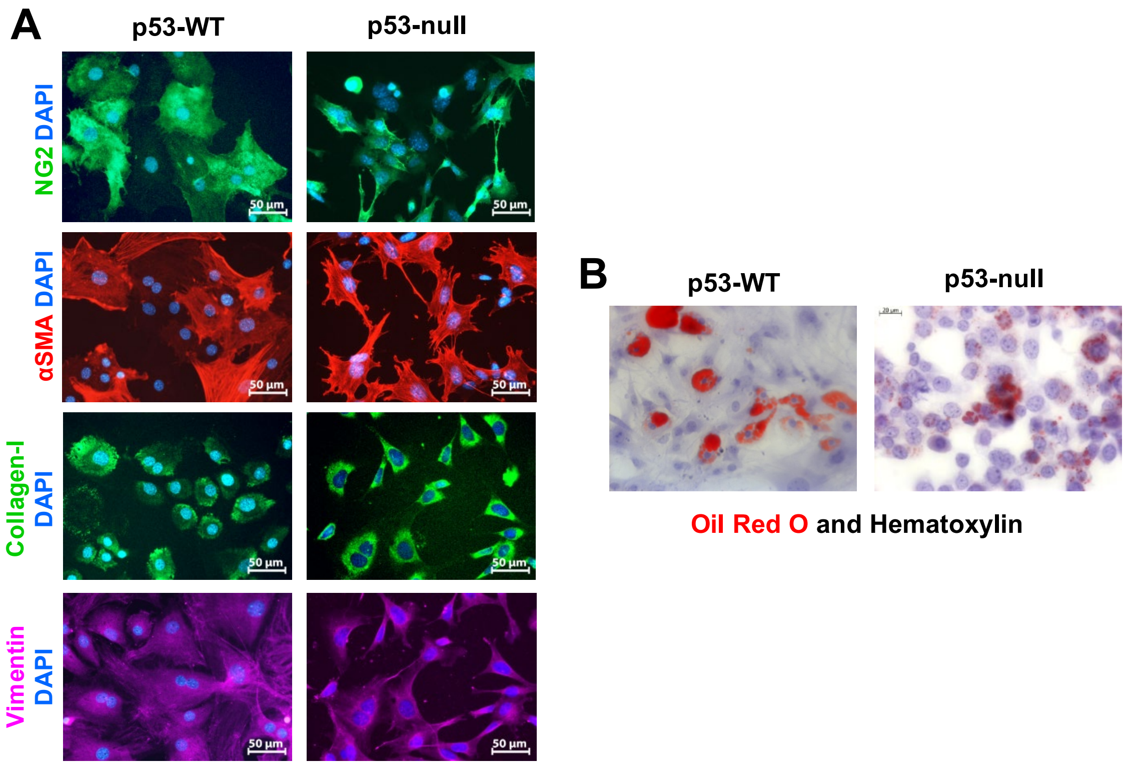

Fig. S5. Characteristics of bone-adherent mesenchymal cells (BAMC) from p53-WT and p53-null mice. BAMC were isolated from control (non-irradiated) p53-WT and p53-null mice and cultivated in vitro for three passages. (A) Immunofluorescent staining was performed for pericyte markers (αSMA and NG2) and fibroblast markers (vimentin, collagen type I). DAPI was used to visualize nuclei. (B) BAMC were treated with adipogenic supplement from the Mouse Mesenchymal Stem Cell Functional Identification Kit and stained with hematoxylin and Oil Red O for the detection of adipocytes.

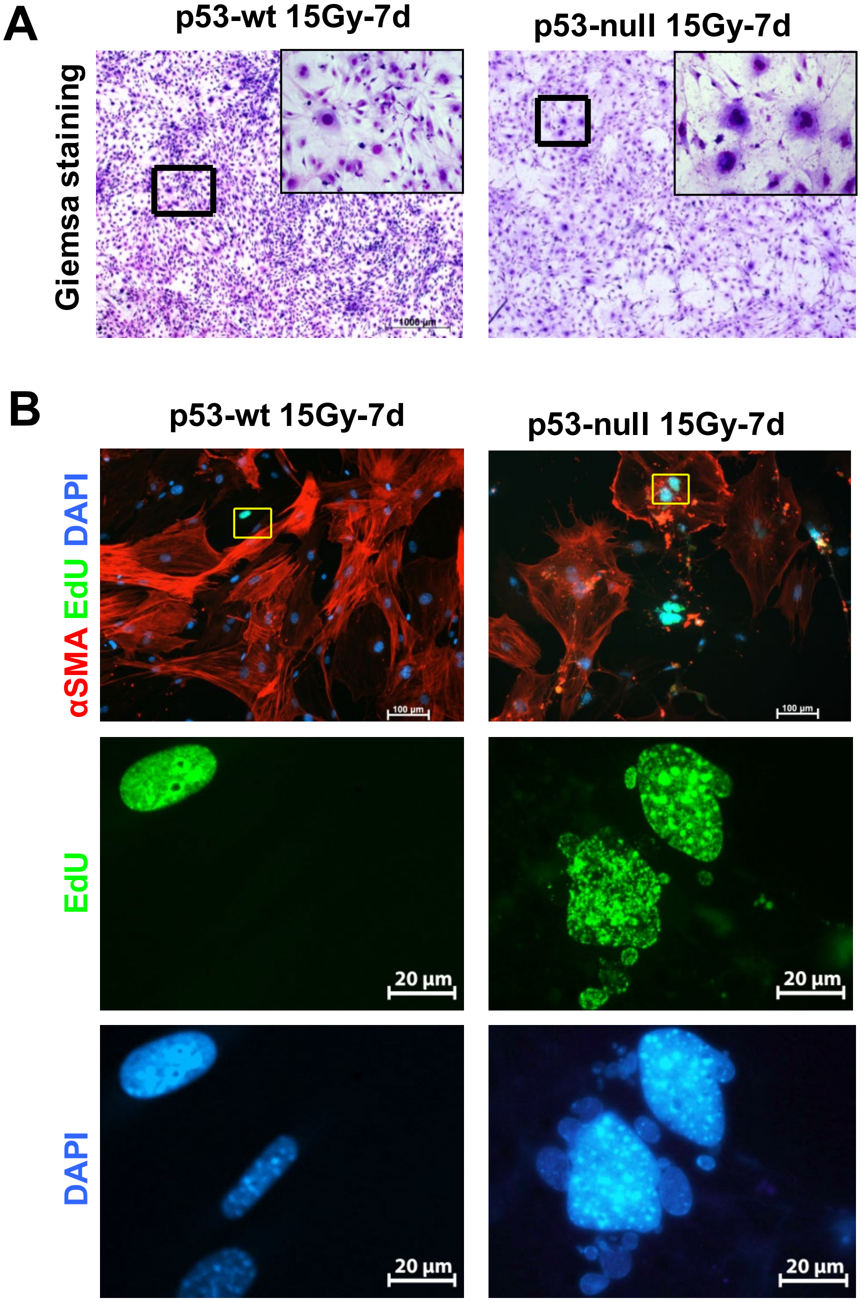

Fig. S6. Nuclei of BAMC from p53-null mice show more morphological signs of damage after in vitro irradiation than those from p53-WT mice. Cultures of BAMC from p53-WT and p53-null mice were irradiated (15 Gy) and analyzed by Giemsa staining (A) and immunofluorescence for αSMA, EdU and DAPI (B) 7 days later. EdU was added 1 h before staining to detect cell proliferation and DAPI was used as a costain to visualize nuclei. Boxed areas of images are shown at higher magnification in inset panel (Giemsa staining) or in lower panels (immunofluorescent staining).

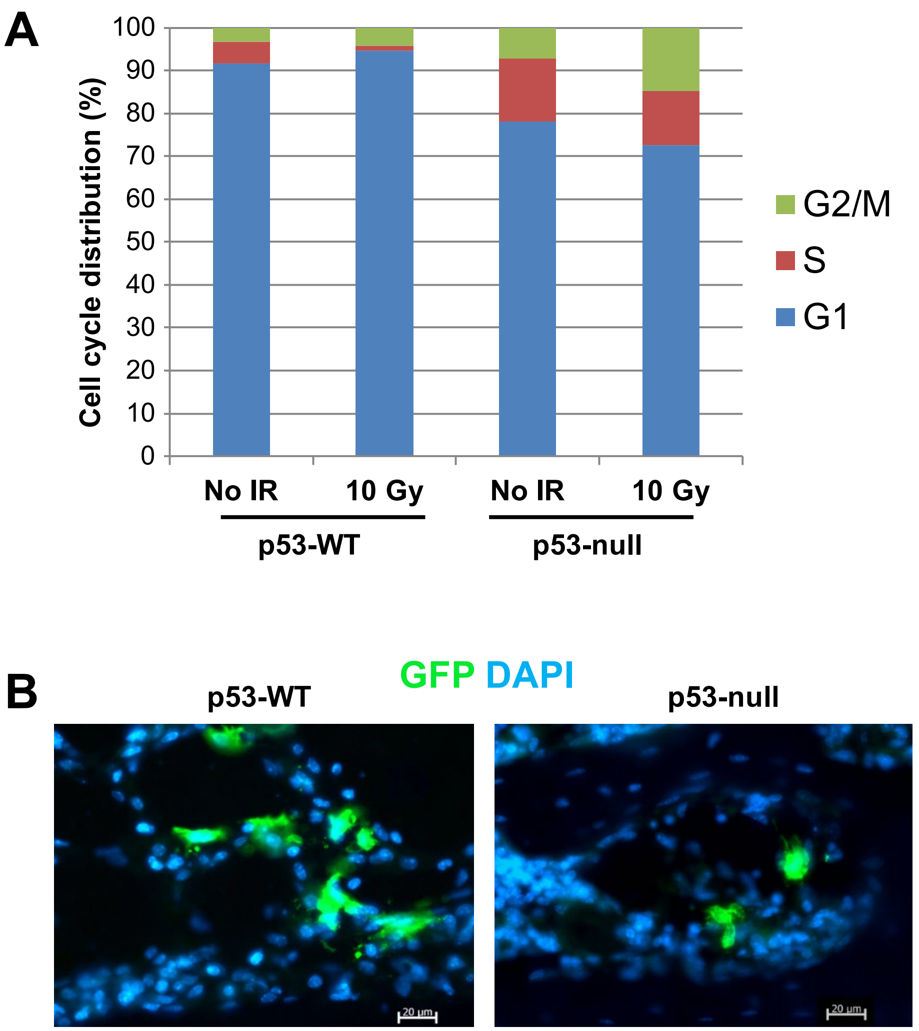

Fig. S7. (A) Analysis of distribution of p53-WT and p53-null irradiated BAMC among the phases of cell cycle. BAMC were isolated from the femurs of 6-week-old WT and p53-null mice (n=10) as described (78). Growing cells were irradiated with 10 Gy (second passage) and 24 hours later analyzed by FACS using propidium iodide staining. Relative distribution of cells among G1, S, and G2/M phases of the cell cycle is shown. (B) Donor GFP-positive BAMC can be detected in BM of recipient mice after co-transplantation with BM cells. BAMC were isolated from GFP-positive transgenic mice (p53-WT) and expanded in culture. 3x105 second-passage BAMC were co-transplanted with 5x106 p53-WT BM cells (numbers are given per mouse) to irradiated p53-WT and p53-null mice that received 13 Gy TBI. 6 days after BMT, longitudinal sections of femoral BM from p53-WT and p53-null mice were analysed for GFP expression.

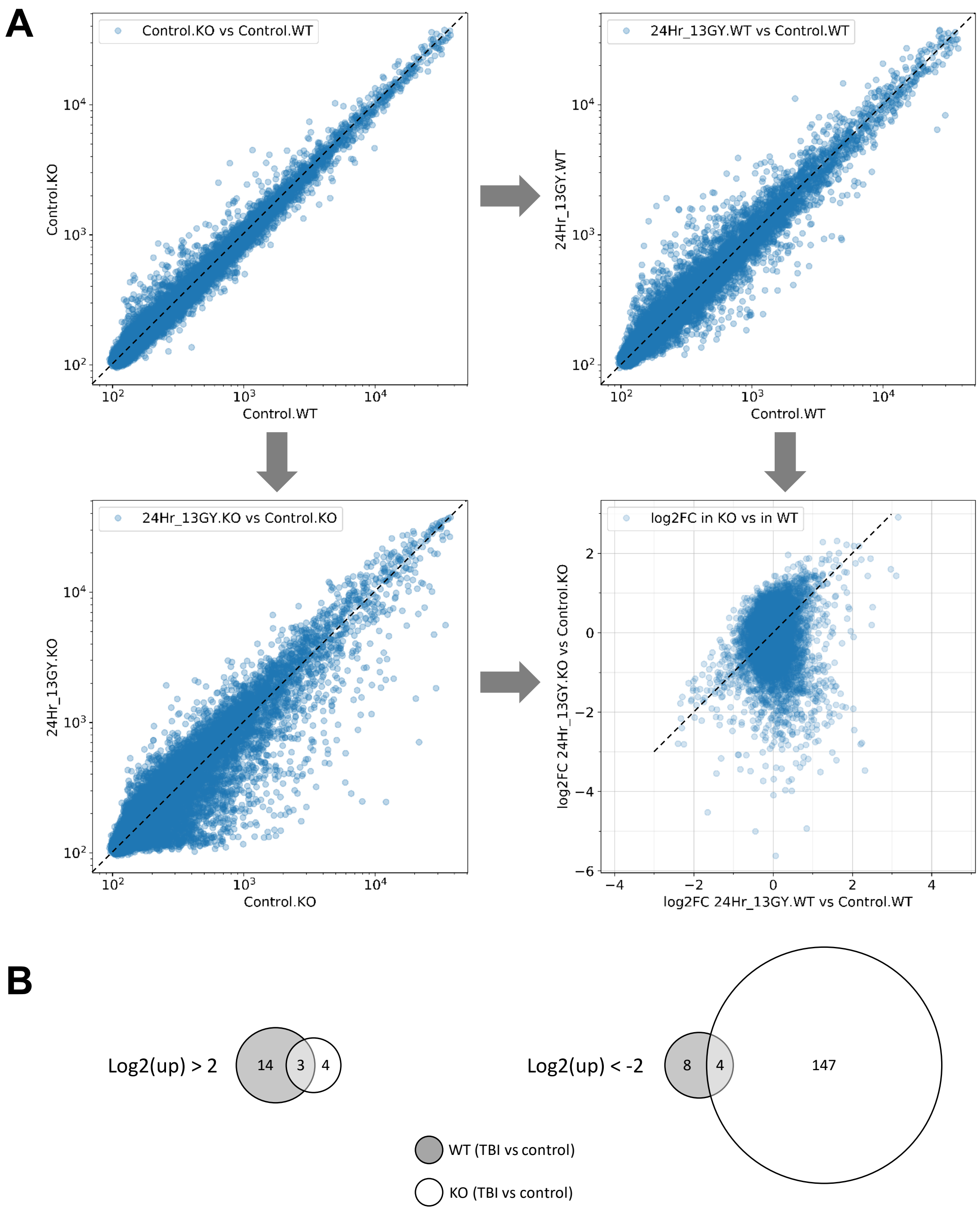

Fig. S8. Changes in mRNA expression levels in BAMC isolated 24 hours post 13 Gy TBI of p53-WT and p53-null mice. Gene expression profiling was conducted using Illumina mouse WG6 microarray hybridization. (A) Scatter plots are shown for: gene expression levels in non-irradiated p53-WT vs non-irradiated p53-null mice (upper left panel), non-irradiated vs irradiated p53-WT mice (upper right panel), non-irradiated vs irradiated p53-null mice (lower left panel), >log2-fold changes (log2FC) of mRNA expression between non-irradiated and irradiated samples in p53-WT mice versus KO mice (lower right panel). Dashed black lines represent eye-guide for equal expressions or for equal log2FC (lower right panel). (B) Venn diagrams showing most up- (log2FC>2) or down- (log2FC<-2) regulated genes in p53-WT and p53-null mice

Table S1. List of primary antibodies used for immunofluorescence

Antigen	Host	Source	Cat no.	Dilution
Actin, alpha-smooth muscle Cy3™	Mouse	Sigma-Aldrich	C6198	1:1000
Perilipin A/B	Rabbit	Sigma-Aldrich	P1873	1:500
NG2 chondroitin sulfate proteoglican	Rabbit	EMD Millipore	AB5320	1:100
Collagen type I	Rabbit	Abcam	Ab34710	1:100
S100A4 (fibroblast-specific protein 1)	Rabbit	EMD Millipore		1:100
CD45	Rat	BioLegend	103101	1:100
CD146	Rat	BioLegend	134702	1:100
CD105	Rat	EMD Millipore	CBL1358	1:100
Ter119	Rat	BioLegend	116201	1:100
Alexa Fluor® 488 anti-mouse/human CD11b	Rat	BioLegend	101217	1:50
F4/80	Rat	eBioscience	14-4801	1:100
MECA-32	Rat	Developmental Studies Hybridoma Bank		
Leptin R	Goat	R&D	AF497	1:100
SDF1 (CXCL112)	Goat	Santa Cruz	sc-6193	1:100
Vimentin	Goat	EMD Millipore	AB1620	1:100
GFP	Chicken	Aves Labs Inc	GFP-1010	1:1000

Table S2. List of secondary antibodies used for immunofluorescence (all secondary antibodies were Donkey Whole IgG affinity purified cross-adsorbed antibodies)

Antibody	Source	Cat no.	Dilution
Anti-rabbit IgG AlexaFluor488	Jackson ImmunoResearch	711-545-152	1:1000
Anti-rabbit IgG Cy5	Jackson ImmunoResearch	711-175-152	1:1000
Anti-rat IgG AlexaFluor 488	Jackson ImmunoResearch	712-545-153	1:1000
Anti-rat IgG AlexaFluor 647	Jackson ImmunoResearch	712-605-153	1:1000
Anti-goat IgG AlexaFluor 488	Jackson ImmunoResearch	705-545-147	1:1000
Anti-goat IgG AlexaFluor Cy5	Jackson ImmunoResearch	705-175-147	1:1000
Anti-chicken IgY AlexaFluor 488	Jackson ImmunoResearch	703-545-155	1:1000
